# Contextual interference during adaptation to asymmetric split-belt treadmill walking results in transfer of unique gait mechanics

**DOI:** 10.1242/bio.028241

**Published:** 2017-11-24

**Authors:** Jacob W. Hinkel-Lipsker, Michael E. Hahn

**Affiliations:** 1 Department of Kinesiology, California State University, Northridge, CA 91330, USA; 2 Department of Human Physiology, University of Oregon, Eugene, OR 97403, USA

**Keywords:** Gait adaptation, Biomechanics, Variable practice, Motor behavior

## Abstract

When humans make errors in stepping during walking due to a perturbation, they may adapt their gait as a way to correct for discrepancies between predicted and actual sensory feedback. This study sought to determine if increased contextual interference during acquisition of a novel asymmetric gait pattern would change lower-limb mechanical strategies generalized to different walking contexts. Such knowledge could help to clarify the role of contextual interference in locomotor adaptation, and demonstrate potential use in future gait rehabilitation paradigms. One belt on a split-belt treadmill was driven at a constant velocity while the other was driven at changing velocities according to one of three practice paradigms: serial, random blocked, or random training. Subjects returned to complete one of two different transfer tests. Results indicate that during acquisition, random practice requires unique gait mechanics to adapt to a challenging walking environment. Also, results from one transfer test close to that of the acquisition experience did not seem to demonstrate any contextual interference effect. Finally, random blocked practice resulted in highly unique changes in step length symmetry on a second, more challenging, transfer test. This perhaps indicates that a moderate level of contextual interference causes unique locomotor generalization strategies.

## Abstract (precis)

**Summary:** Specific biomechanical gait adaptations occur as a result of variance of stepping errors during walking. Humans optimize a novel gait pattern based on different levels of error variance.

## INTRODUCTION

Locomotion is a task that humans can adapt rapidly in an environment that demands a change in lower limb mechanical function. Generally, a locomotor adaptation serves as a means by which the central nervous system (CNS) can minimize a specific cost of walking, such as energy expenditure, balance, or pain (Bastian, 2008; Todorov, 2004). Also, this adaptation can reflect flexibility within the locomotor control system that allows humans to maintain walking performance in the face of new or difficult conditions. While flexibility of gait mechanics may manifest as a permanently different pattern after unilateral lower-limb amputation (Sanderson and Martin, 1997) or hemiparetic stroke (Olney and Richards, 1996), it has also been reported to be acutely observable within the first 12-15 strides of a new walking context in able-bodied individuals (Prokop et al., 1995). Additionally, biomechanical gait adaptations can occur as a response to uneven terrain (Voloshina et al., 2013), or physical constraints such as an active exoskeleton (Banala et al., 2010).

The cerebellum may be responsible for this rapid adaptability. During locomotion, stepping errors can occur when the walking environment changes. This causes a discrepancy between expected and actual proprioceptive feedback that is dynamically detected by the cerebellum (Morton and Bastian, 2007), which then corrects for the discrepancy by directly overriding the basic locomotor rhythm provided by spinal pattern generators (Takakusaki, 2013). The cerebellum may also be responsible for updating a feed-forward model as a result of this sensory discrepancy, which is then relayed to premotor cortical areas for updating of the motor plan for locomotion (Blakemore et al., 2001; Galea et al., 2011; Seidler et al., 2013). Therefore, stepping errors during locomotion may be directly responsible for how an individual adapts to a novel gait pattern, as they can be immediately corrected and more permanently planned for through separate processes.

Researchers have made note of this effect during observation of human adaptation to asymmetric split-belt treadmill walking (SBW), an experimental paradigm where two belts on a treadmill are driven at different velocities as a method for inducing a gait asymmetry. For able-bodied individuals who typically walk symmetrically, this method can be viewed as a way to introduce a novel context for walking. As these individuals walk in the novel context, different adaptation strategies can be observed over time. During this process, the size of stepping errors encountered during walking seems to affect how individuals adapt their gait pattern. For example, Torres-Oviedo and Bastian (2012) proposed that the distribution of errors incurred during adaptation to SBW ultimately affects the transfer, or generalizability, of an asymmetric walking pattern to overground walking. Also, Sawers and Hahn (2013) and Sawers et al. (2013a,b) have discussed how a gradual introduction of asymmetry (where the belt velocity for one limb increases walking asymmetry over time) leads to better retention and transfer of limb endpoint control and mediolateral balance control compared to a large, sudden introduction of asymmetry (which abruptly induces large stepping errors).

Broadly, sensory prediction errors have been discussed in the context of motor learning for decades. Shea and Morgan (1979) were among the first to introduce the idea of contextual interference as a way to increase motor learning, where environmental conditions during practice are ordered in a noisy fashion to intentionally induce sensory prediction errors. When contextual interference is used as a training tool for learning of a motor skill, it creates an unpredictability that requires the learner to solve for sensory prediction errors instead of solely predicting the required movement parameters for optimal task performance (Lee and Magill, 1983). As such, noisy environmental conditions may induce a trial-and-error learning mechanism, where unpredictable sensory feedback ultimately drives the need for individuals to explore the space of potential learning solutions (Wu et al., 2014). Indeed, even in simulations, a noisy optimization algorithm can increase the rate of learning in artificial neural networks (Burton and Mpitsos, 1992), highlighting an adaptive learning process that may be applicable to biological systems.

In humans, the method of learning referred to as variable practice has been demonstrated to be an effective tool for the acquisition of novel motor skills, ranging from bimanual coordination tasks (Tsutsui et al., 1998) to basketball shooting (Landin et al., 1993). In addition, it has been used effectively as a means to refine already-acquired motor skills or increase their generalizability to conditions outside of the practice context, with an increased ability for an individual to scale movement parameters following variable practice. Specifically, a positive learning effect of variable practice has been noted in reaching and grasping tasks (Hanlon, 1996; Krakauer, 2006; Turnham et al., 2012) and stepping following a hemiparetic stroke (Pollock et al., 2014), indicating its potential for use in rehabilitative settings.

It remains largely unknown how variable practice influences human locomotor behavior when individuals are asked to adapt their walking pattern to novel contexts. While the studies previously mentioned by Torres-Oviedo and Bastian (2012), Sawers and Hahn (2013) and Sawers et al. (2013a,b) investigate the effect of sensory prediction errors on locomotor adaptation during SBW, the same belt was always driven at a faster velocity than the other. This may have allowed some predictability of sensory feedback and motor parameters. Recent work has shown how, during locomotor adaptation, individuals seem to organize the roles of the two lower limbs into a slow limb and fast limb – with each exhibiting different mechanical output (Ogawa et al., 2014). One way to further increase the amount of noise in the adaptation experience may be to vary the roles of the limbs to prevent one from moving at a consistently fast velocity, thereby preventing the assignment of limb roles. To address this, we have recently introduced a SBW paradigm where treadmill belt velocities on one limb move both faster and slower than the other limb. These velocities differed according to specific practice paradigms. We observed that increased contextual interference in belt velocities on one limb led to better transfer of mediolateral balance control, but only to an extent; too much contextual interference resulted in less transfer as seen through worsened mediolateral balance control (Hinkel-Lipsker and Hahn, 2017).

While the studies discussed in the previous paragraph do demonstrate that increased stepping variability may influence locomotor adaptation to some degree, they all utilized task-related performance variables as their measures of learning. In a case where a new rehabilitative approach for gait is implemented, it may be necessary to know how individuals adapt beyond performance outcomes. An analysis of lower limb kinematics and kinetics, in conjunction with the previous work done in this area of research, would thereby clarify if improved gait adaptation with regards to performance metrics is accompanied by gait mechanics that are also indicative of increased walking performance. In addition, lower-limb mechanics can also improve the resolution of these analyses, as kinematic and kinetic measures can help to further clarify the walking strategy adopted by able-bodied subjects during acquisition and transfer tests. To date, such a study has not been done, and it is unknown if individuals adopt different mechanical strategies as a result of higher levels of contextual interference during acquisition of a novel, asymmetric gait.

In this study, we explored how three different levels of contextual interference affected lower limb mechanics during acquisition and transfer of a novel asymmetric gait. Subjects in the group with the lowest level of contextual interference in this study, serial practice, were given a linear increase in belt velocity on their dominant limb during acquisition. The next highest level, random blocked practice, involved a random change in belt velocity every 20 strides on the dominant limb. The highest level, random practice, invoked a random change in belt velocity every stride on the dominant limb. Since random practice was most difficult during acquisition, we hypothesized that (1) this group would have significantly different lower-limb mechanics compared to the other practice groups and baseline, symmetric walking. In our previous work, we found that random blocked practice had significantly better control of mediolateral balance during a transfer test (Transfer 1) that introduced an asymmetry close to what was experienced during the acquisition phase compared to the other practice groups (Hinkel-Lipsker and Hahn, 2017). It is likely, then, that this group found a unique mechanical walking solution as a result of this practice paradigm. Therefore, we hypothesized that (2a) early in Transfer 1, individuals in the random blocked practice group would exhibit gait mechanics significantly different from baseline, symmetric walking and the other two practice groups. However, we also hypothesized that (2b) by the end of Transfer 1 all practice groups will have become fully adapted to the asymmetric pattern, as demonstrated by gait mechanics that were not significantly different from each other. This would be demonstrative of the two limbs self-organizing into roles of one ‘fast’ limb, and one ‘slow’ limb (Ogawa et al., 2014; Roper et al., 2017). Finally, early in a second transfer test where the belt asymmetry was much different from what was experienced during acquisition (Transfer 2), we hypothesized that (3a) serial practice would result in significantly different gait mechanics compared to the other two groups and baseline, symmetric walking. This effect would be due to the lower level of contextual interference that this group experienced during acquisition, limiting their ability to generalize their pattern to a walking environment much different than acquisition. Much like Transfer 1, we also hypothesized that (3b) by the end of Transfer 2, there would be no significant difference in gait mechanics between practice groups.

## RESULTS

**Fig. 1.**
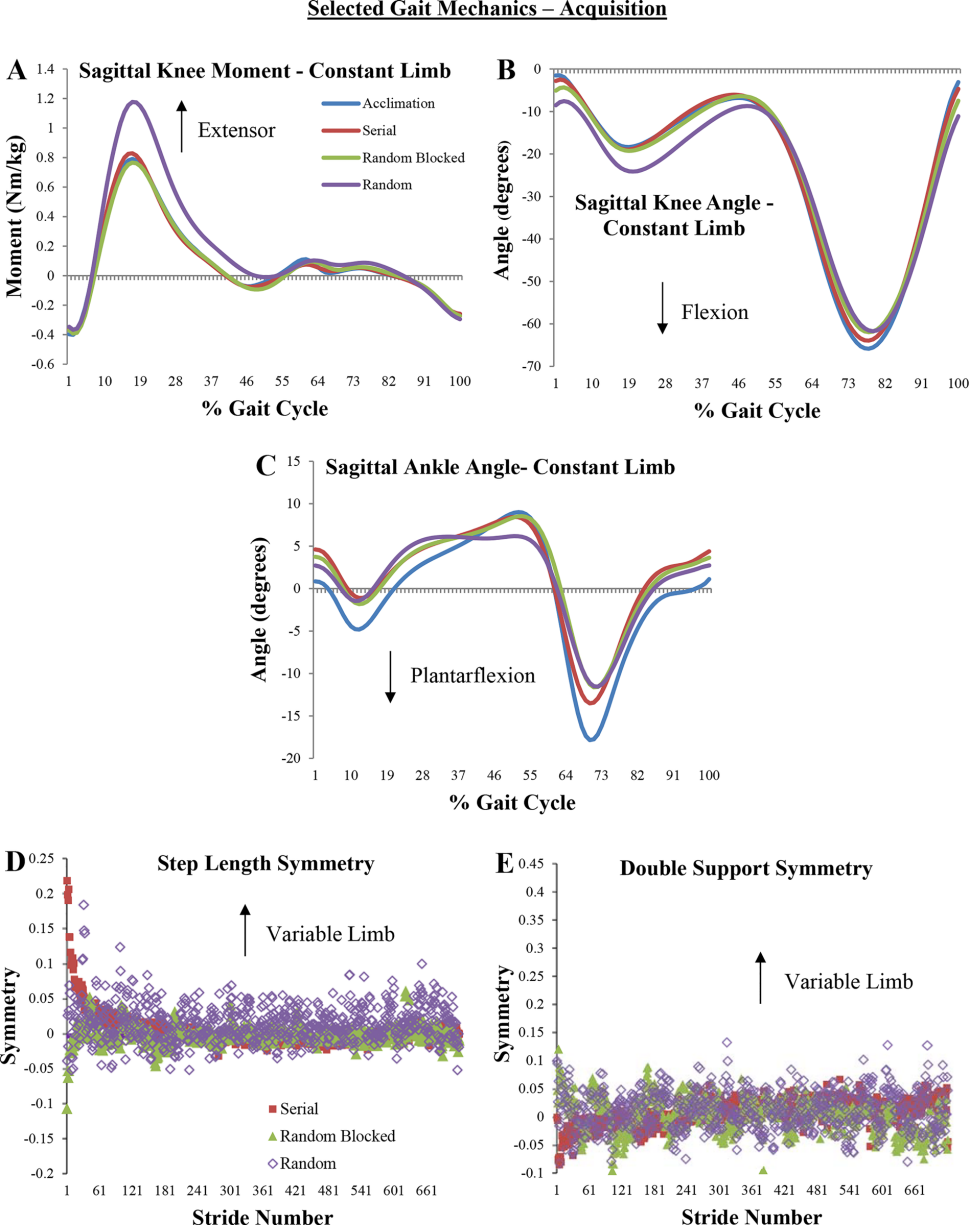
**Selected gait mechanics from the acquisition phase.** (A-C) Ensemble-averaged curves from each subject in each practice group. Acclimation curves represent symmetric walking by all subjects in all practice groups. (D,E) Averaged stride-by-stride symmetry values from each subject in each practice group.

For acquisition, a MANOVA revealed a significant main effect of limb (*F*=3.842, *P<*0.001, 
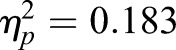
) and group (*F*=4.119, *P*<0.001, 
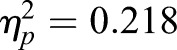
), and a significant limb×group interaction (*F*=1.483, *P<*0.05, 
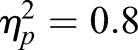
). Pairwise comparisons indicate that random practice had a significantly greater peak hip extensor moment (HEM) during swing on the constant limb compared to acclimation (Table 1), and on the variable limb compared to acclimation, serial, and random blocked practice (Table 1). The random practice group also walked with a significantly greater knee extensor moment (KEM) and knee flexion angle (KFA) during stance on the constant limb compared to the other two groups and acclimation (Table 1, Fig. 1A,B). This group also walked with a significantly shorter step length on both limbs (Table 1) compared to all groups.

**Table 1.**
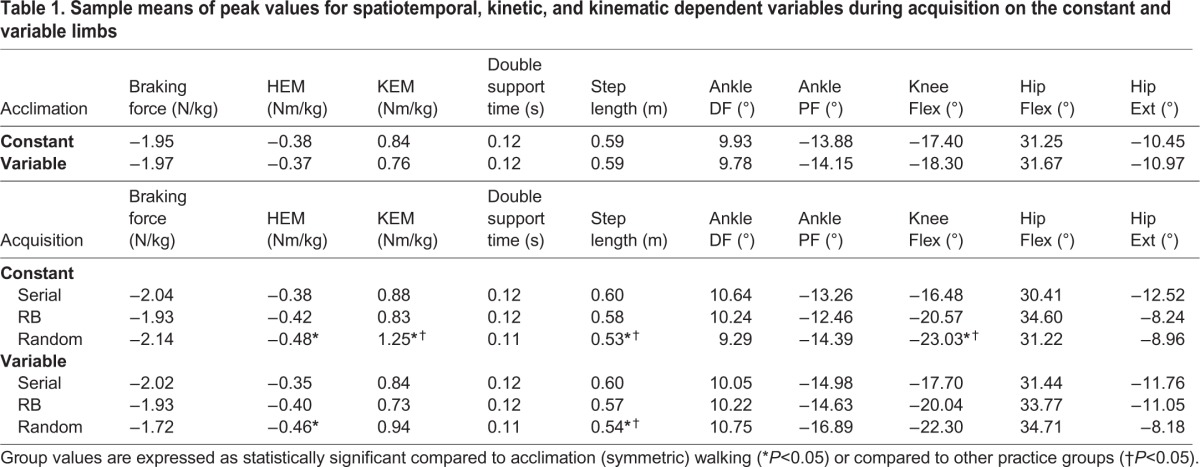
**Sample means of peak values for spatiotemporal, kinetic, and kinematic dependent variables during acquisition on the constant and variable limbs**

During early Transfer 1, there was a significant main effect of limb (*F*=16.601, *P*<0.001, 
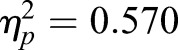
) and group (*F*=4.881, *P*<0.001, 
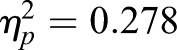
), and a significant limb×group interaction (*F*=3.029, *P*<0.001, 
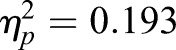
). An examination of pairwise comparisons revealed that all groups had a significantly greater HEM during swing on the constant limb compared to acclimation, and all but the random blocked practice group showed the same effect on the variable limb. Random practice had a significantly higher KEM on the constant limb compared to acquisition, while the random blocked practice group's KEM was significantly higher than acclimation on the variable limb. Additionally, all groups had a significantly shorter double support time on both limbs compared to acclimation, and significantly greater plantar flexion during late stance on the variable limb compared to acclimation (Table 2).

**Table 2.**
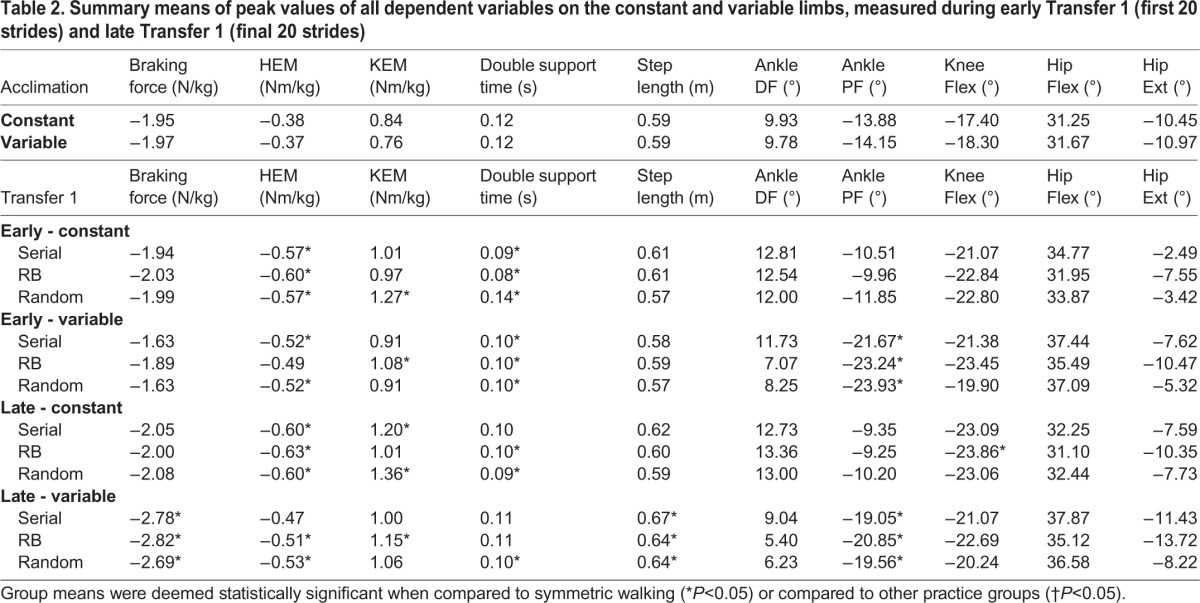
**Summary means of peak values of all dependent variables on the constant and variable limbs, measured during early Transfer 1 (first 20 strides) and late Transfer 1 (final 20 strides)**

**Fig. 2.**
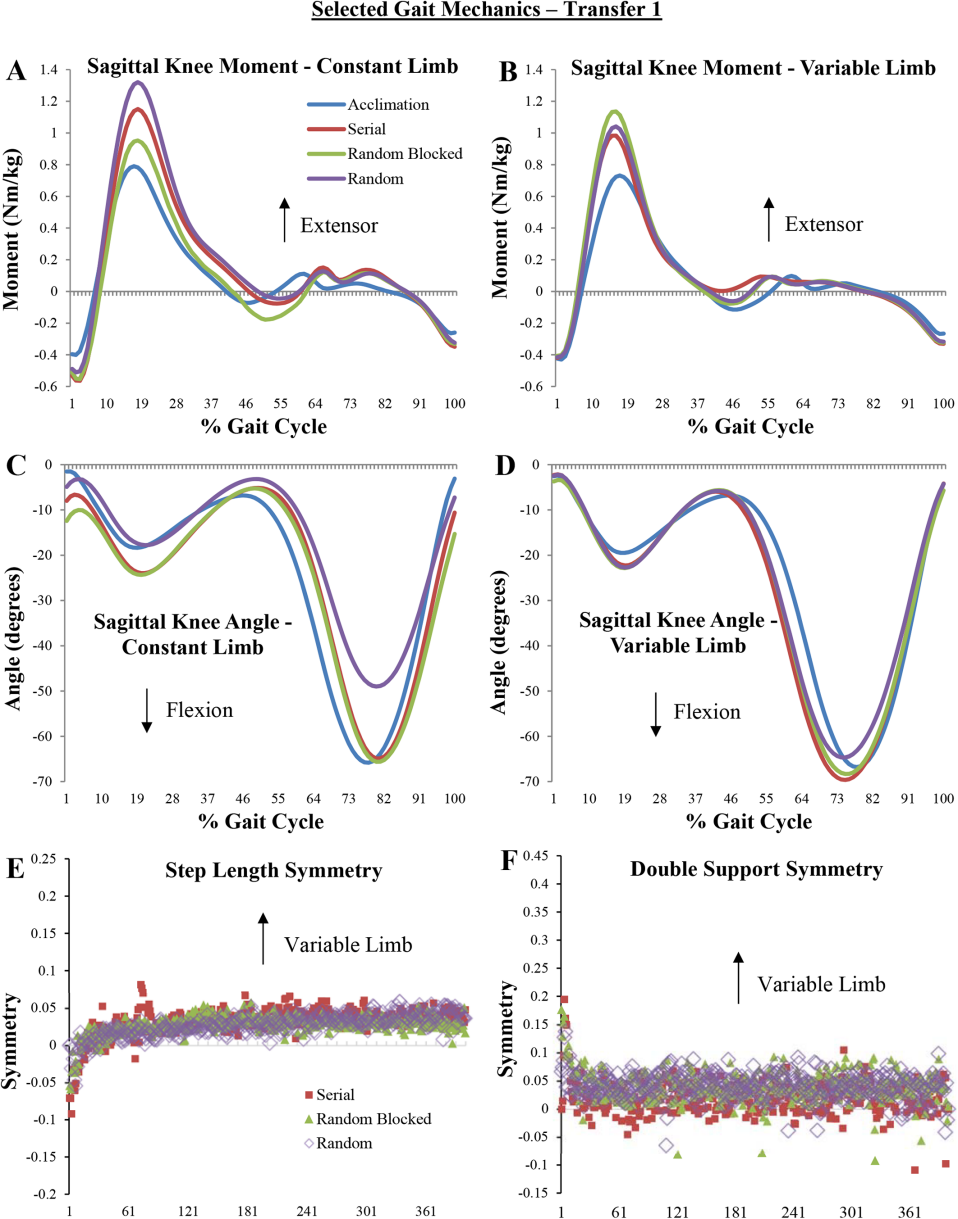
**Selected gait mechanics from Transfer 1 phase, where subjects walked at a 1.5:1 (variable limb:constant limb) asymmetry.** (A-D) Ensemble-averaged curves from each subject in each practice group. Acclimation curves represent symmetric walking by all subjects in all practice groups. (E,F) Averaged stride-by-stride symmetry values from each subject in each practice group.

A MANOVA for the late Transfer 1 analysis window showed a significant main effect of limb (*F*=15.163, *P*<0.001, 
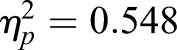
) and group (*F*=3.628, *P*<0.001, 
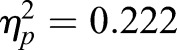
), and a significant limb×group interaction (*F*=2.728, *P*<0.001, 
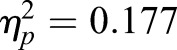
). Pairwise comparisons showed a significantly greater peak braking force for all groups on the variable limb compared to acclimation, and a significantly greater HEM on the constant limb compared to acclimation. For the variable limb, however, only the random blocked and random groups walked with a significantly greater HEM compared to acclimation. Subjects in the serial and random groups walked with a significantly greater KEM compared to acclimation on the constant limb (Table 3, Fig. 2A), while only the random blocked practice group had a significantly greater KEM than acclimation on the variable limb (Table 3, Fig. 2B). The random blocked and random groups had a significantly greater double support time than acclimation on the constant limb, while only random practice resulted in a significantly greater double support time on the variable limb compared to acclimation. All groups had a significantly shorter step length on the variable limb compared to acclimation, significantly less plantar flexion during late stance, and the random blocked practice group had a significantly greater KFA on the constant limb compared to acclimation (Table 3, Fig. 2C).

**Table 3.**
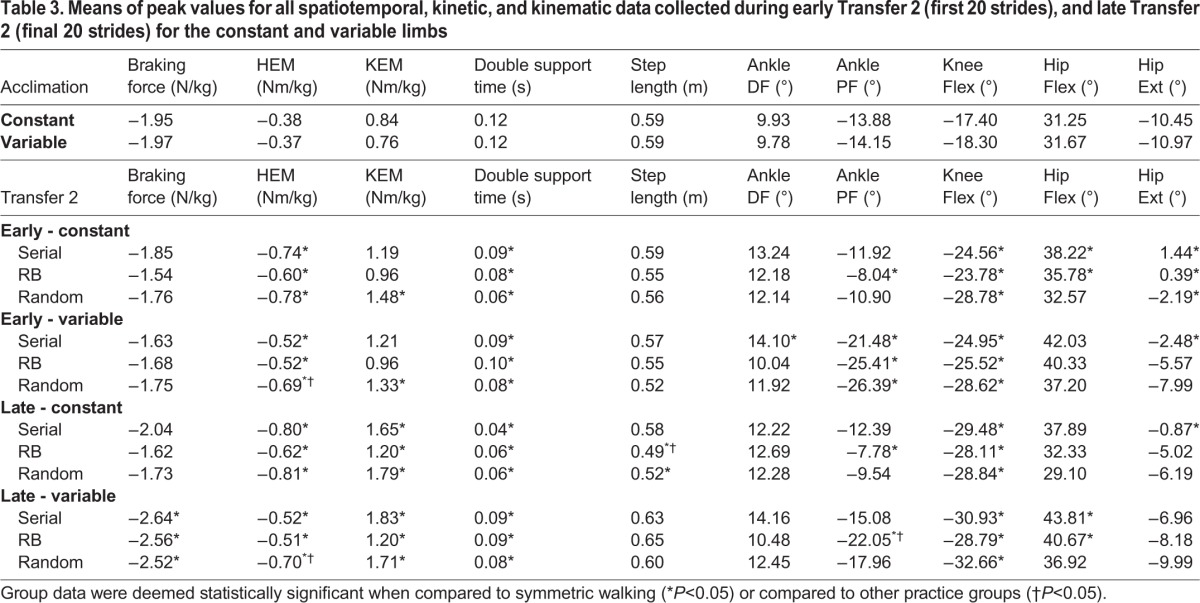
**Means of peak values for all spatiotemporal, kinetic, and kinematic data collected during early Transfer 2 (first 20 strides), and late Transfer 2 (final 20 strides) for the constant and variable limbs**

During early Transfer 2, a significant main effect of limb (*F*=21.704, *P*<0.001, 
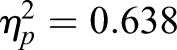
), group (*F*=6.506, *P*<0.001, 
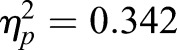
), and a significant limb×group interaction (*F*=3.598, *P*<0.001, 
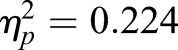
) was noted. On both limbs, all groups had a significantly higher HEM during swing compared to acclimation, and random practice resulted in a significantly higher HEM compared to serial practice on the variable limb. The random practice group also demonstrated a significantly greater KEM during stance on both limbs compared to acclimation. All groups had a significantly shorter double support time compared to acclimation on both limbs. The serial practice group had a significantly greater peak ankle dorsi-flexion angle (ADA) during stance compared to acclimation on the constant limb, while the random blocked practice group had a significantly greater ankle plantar-flexion angle (APA) on the constant limb compared to acclimation, and all groups had significantly less plantar flexion during late stance compared to acclimation on the variable limb. Moreover, all groups walked with significantly greater knee flexion in both limbs during stance, and with significantly less hip extension during stance on the constant limb. However, only the serial practice group had significantly less hip extension during stance on the variable limb compared to acclimation. Additionally, the serial and random blocked practice groups had a significantly greater peak hip flexion angle (HFA) during swing compared to acclimation (Table 3).

**Fig. 3.**
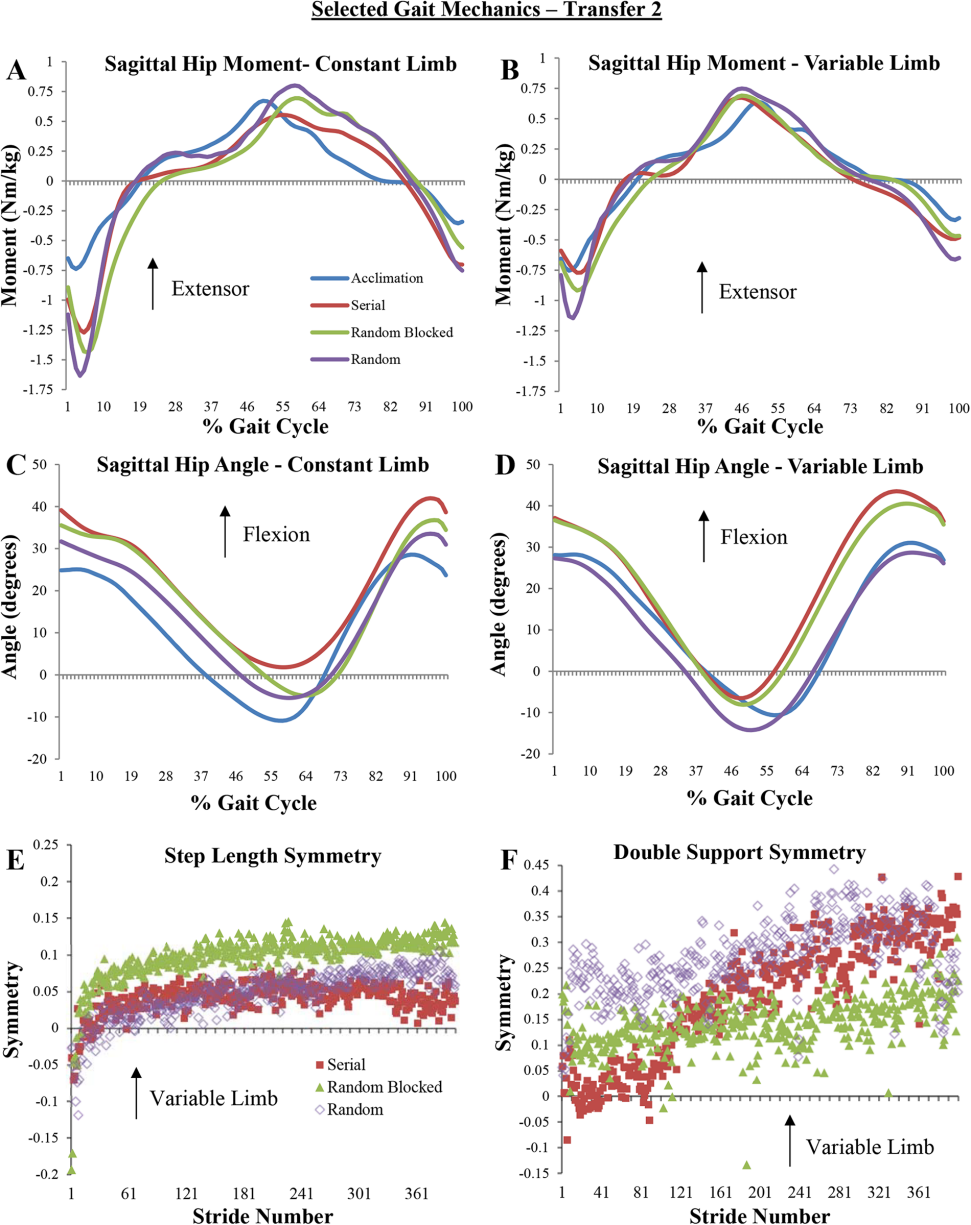
**Selected gait mechanics from Transfer 2 phase, where subjects walked at a 2:1 (variable limb:constant limb) asymmetry.** (A-D) Ensemble-averaged curves from each subject in each practice group. Acclimation curves represent symmetric walking by all subjects in all practice groups. (E,F) Averaged stride-by-stride symmetry values from each subject in each practice group.

Finally, the late Transfer 2 MANOVA also demonstrated a significant main effect of limb (*F*=16.050, *P*<0.001, 
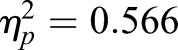
) and group (*F*=6.204, *P*<0.001, 
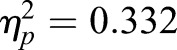
), and a significant limb×group interaction (*F*=3.371, *P*<0.001, 
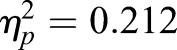
). These main effects are explained by multiple pairwise comparisons. On the variable limb, all groups had a significantly greater peak braking force compared to acclimation, and a significantly greater HEM compared to acclimation on both limbs. Additionally, serial and random blocked practice resulted in a significantly lower peak HEM compared to random practice (Table 3, Fig. 3A,B). All groups had a significantly greater KEM on both limbs compared to acclimation, as well as a significantly shorter double support time on both limbs. On the constant limb, the random blocked and random practice groups had a significantly shorter step length compared to serial practice and acclimation. Also, the random blocked practice group had a significantly greater amount of APF during late stance compared to acclimation on both limbs, and all groups had significantly greater knee flexion on both limbs compared to acclimation. Finally, compared to acclimation, the serial practice group walked with a more flexed hip throughout the gait cycle, as evidenced by a significantly lower peak hip extension angle during stance on the constant limb (Table 3, Fig. 3C) and a significantly greater hip flexion angle during swing on the variable limb (Table 3, Fig. 3D). The random blocked practice group also demonstrated a significantly greater hip flexion angle during swing on the variable limb compared to acclimation (Table 3).

## DISCUSSION

These results indicate that the cohort in this study utilized unique gait strategies to accommodate the novel practice experience, and it seems that these adopted practice strategies resulted in different strategies for generalization of gait to a novel context. While the results do demonstrate the adoption of novel mechanics, they do not offer an explanation as to why these mechanical adaptations occurred. Therefore, this discussion is framed in the context of group differences, but we have specifically avoided further reasoning as to why these mechanical adaptations occurred, as it was considered overly speculative at this time. Since some adaptations likely occurred as a result of walking asymmetrically in general and not due to a specific acquisition experience, they are also not discussed.

The results from the acquisition phase largely support Hypothesis 1. Overall, it seems that the unpredictability associated with random practice resulted in different gait mechanics relative to symmetric walking during acclimation and the other two groups during this phase. For example, the random practice group had a significantly greater HEM during swing on both limbs compared to acclimation. On the one hand, while increased HEM is related to greater hamstring activation in an attempt to slow the velocity of the swing limb prior to foot contact (Winter and Rogers, 1992), further research quantifying muscle activity during this task is needed to clarify this strategy. Also, since there was no difference in HEM between the random practice group and the other two groups, the degree to which contextual interference influences this variable is unknown. On the other hand, the random practice group had a significantly greater peak knee extensor moment (Table 1, Fig. 1A) and knee flexion angle (Table 1, Fig. 1B) during stance on the constant limb compared to acclimation and the other two practice groups. While not statistically significant, this group also did not have an increase over time in ankle dorsiflexion during stance (Fig. 1C), which was visible for acclimation and the other two practice groups. This may be indicative of a walking pattern similar to that of wearing a ski boot or rigid ankle-foot orthosis, where ankle range of motion is limited at the expense of greater knee flexion (Abel et al., 1998; Radtka et al., 2005), and thereby an increased KEM is needed to maintain support of body weight.

We have previously shown that random practice results in a significantly greater challenge to mediolateral balance control during acquisition (Hinkel-Lipsker and Hahn, 2017). Therefore, the lack of increasing ankle dorsiflexion and significantly greater knee flexion angle and knee extensor moment during stance on the constant limb may be demonstrative of a potential strategy to limit forward propulsion onto the variable limb. While this strategy cannot be further quantified from the results of this study, previous work has discussed how ankle dorsiflexion during stance facilitates storage and return of strain energy to propel the limb forward during late stance (Don et al., 2007; Orendurff et al., 2005). In comparison, the serial and random blocked practice groups did not demonstrate any mechanical adaptations that were significantly different from each other or compared to symmetric walking (Table 4). This may be indicative of the relative ease subjects had in adapting their gait during these paradigms.

**Table 4.**
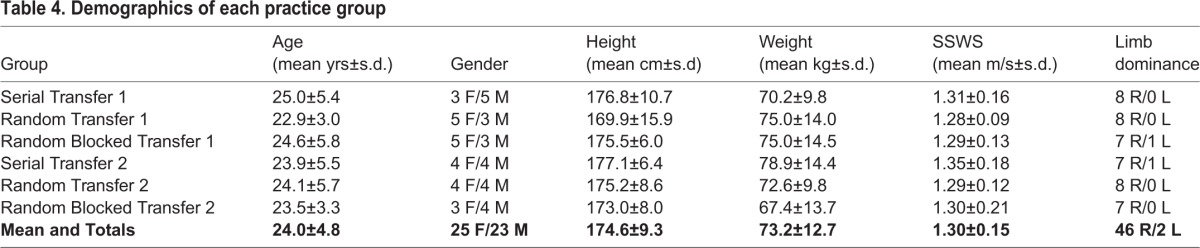
**Demographics of each practice group**

Finally, during acquisition the serial practice group began with a large step length asymmetry biased towards the variable limb (i.e. they utilized a longer step length on the variable limb), but this was quickly calibrated and was relatively symmetrical for most of this phase, even with a continuously increasing belt velocity on the variable limb (Fig. 1D). This pattern of adaptation is consistent with previous split-belt walking research (e.g. Roemmich and Bastian, 2015). On the other hand, the random blocked and random practice groups had widely variable step length symmetry patterns that did not appear to converge towards symmetry over time. This effect is not surprising, since both groups underwent large, random changes in belt velocity during this phase. Other than a more variable pattern for the random blocked and random groups, very few group trends in double support symmetry are apparent (Fig. 1F).

For the first 20 strides of the Transfer 1 test, subjects in all groups exhibited gait mechanics that were significantly different from symmetric walking, but not from each other (Table 2). These adaptations are likely the result of an asymmetric gait in general, and not a unique strategy adopted due to a specific practice experience. Hence, Hypothesis 2a is not supported by these results. Similarly, at the end of the Transfer 1 bout, some generalization strategies were apparent but there were no significant differences between practice groups. One notable change from symmetric walking that persisted until the end of the transfer test was that the serial and random practice groups had a significantly greater knee extensor moment during stance on the constant limb compared to symmetric walking (Fig. 2A), while the random blocked practice group had a significantly greater knee extensor moment during stance on the variable limb compared to symmetric walking (Fig. 2B). On both limbs, all group difference changes occurred without a change in knee flexion angle on the same limb compared to symmetric walking (Fig. 2C,D), potentially indicating an increase in knee stiffness on the limb in question (DeVita and Skelly, 1992). This asymmetrical loading pattern has been previously observed in patient populations such as those with unilateral knee osteoarthritis (Kumar et al., 2013) or lower-limb amputation (Winter and Sienko, 1988), and may indicate preference of supporting body weight with one limb over the other. However, since these differences between groups were not statistically significant, and Hypothesis 2b is not supported.

The trends in step length and double support symmetry over time indicate little to no group differences (Fig. 2E,F), and reflect symmetric walking across groups from early in the test. Therefore, the data from Transfer 1 demonstrate no effect of practice group. These findings are surprising given our previous findings from this cohort, where random blocked practice had less variability in balance control compared to the other groups (Hinkel-Lipsker and Hahn, 2017). It is possible that since the 1.5:1 belt asymmetry was close to that of the maximum asymmetry experienced during acquisition, all subjects were able to easily generalize their acquired pattern to this new context. Overall, the results from Transfer 1 show that when generalization to a novel context close to that of the practice experience is required, additional contextual interference during practice may not be necessary.

When the transfer context is more different from the practice experience, as was the case with the Transfer 2 test, the contextual interference effect during practice is more apparent. First, the random practice group had a significantly greater hip extensor moment on the variable limb early in the transfer test compared to acclimation and the other two practice groups (Table 1). This strategy persisted throughout the test, as hip extensor moment was significantly greater for the last 20 strides (Table 1, Fig. 3B). No group effect was evident on the constant limb (Fig. 3A). As previously discussed from the acquisition results, this may be related to an inability to swing. Since neither hip swing velocity nor muscle activation of the hip extensors were quantified in this study, more research is needed to confirm this postulation. It is possible that too much contextual interference, in this case, led to an inability to generalize swing phase control of the limb, since this group was unable to find a solution during acquisition due to the random belt velocity changes on the variable limb. While we have previously noted that serial practice has a lesser ability to control mediolateral balance during Transfer 2 (Hinkel-Lipsker and Hahn, 2017), the serial practice group did not exhibit any differential gait mechanics compared to the other two practice groups. Therefore, Hypotheses 2a and 2b are not supported by the results of this study.

Another notable observation from Transfer 2 is that the serial practice group walked with much less hip extension on the constant limb compared to the other two practice groups (Fig. 3C). While this adaptation was not statistically significant, it is noteworthy since, on average, subjects in this group maintained flexed hips during late stance on the constant limb. This effect is not apparent on the variable limb (Fig. 3D). This may represent a method to maintain whole-body center of mass position within the base of support created by the foot in contact with the ground, which may be indicative of instability in the sagittal plane (DeVita and Hortobagyi, 2000). Although we previously noted that serial practice had a high level of mediolateral balance control variability in the frontal plane (Hinkel-Lipsker and Hahn, 2017), no further assumptions with regards to sagittal plane balance can be made from the results of this study as there were no statistically significant differences between practice groups during Transfer 2.

Finally, step length and double support symmetry diverge from greater symmetry to lesser over the course of time during Transfer 2 (Fig. 3E,F). This finding is in contrast to many other split-belt walking studies, where over the course of adaptation individuals tend to move towards more symmetric step lengths and double support times, even with belt velocities remaining constantly asymmetric (e.g. Reisman et al., 2005, 2013; Roemmich and Bastian, 2015). In this study, step lengths and double support times increased on the variable limb relative to the constant limb. In another recent study it has been noted that over the course of a gait adaptation the limbs begin to operate individually, and lower-limb joint kinetics are scaled to values close to that of symmetric walking at the same velocity (Roper et al., 2017). While such a conclusion cannot be drawn from the results of this study, it is possible that over time subjects attempted to match their step lengths to the velocity of the treadmill belts during Transfer 2. Since random blocked practice had a significantly shorter step length compared to the other two groups on the constant limb, it is possible that this level of contextual interference leads to an increased ability to independently scale step lengths to belt velocity. It is unknown whether this adaptation is beneficial or not, however, and thus future research in this area could help to clarify the disparity in results between previous studies and the present work.

**Fig. 4.**
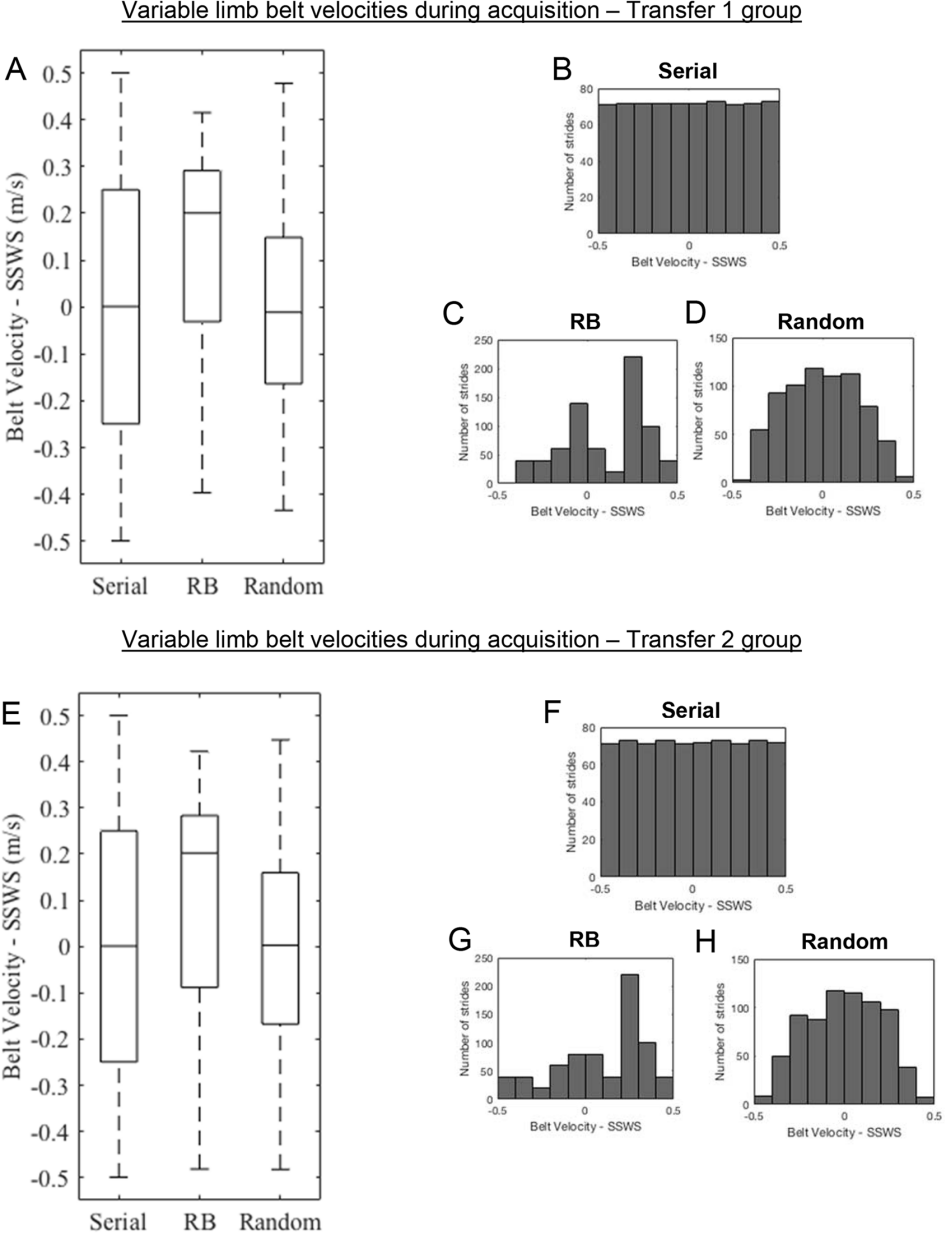
**Ranges and distributions of belt velocities for the variable limb during acquisition.** (A) The ranges for subjects who completed the Transfer 1 test. (B-D) Belt velocity distributions for the serial, random blocked (RB), and random practice groups, respectively, who completed the Transfer 1 test. (E) The ranges for subjects who completed the Transfer 2 test, and (F-H) show the distributions. Box and whisker plots in A and E show median values and distribution ranges for the Transfer 1 and Transfer 2 groups, respectively.

Some limitations may have impacted the results of this study. First, the measured gait parameters were averaged across the entire acquisition experience, which may have moderated the measured effect of some adaptations because the variable limb was at times slower and at times faster than the constant limb. However, group comparisons could not have been made if the acquisition analysis was separated into smaller time windows because the variable limb was not moving at the same velocity for all groups during any given window. Thus the time windows could not be matched for belt velocity because those specific gait asymmetries occurred at different points of time for each group. Analysis of the overall acquisition experience provides a snapshot of the overall strategy adopted by individuals due to variability on one belt, not necessarily a faster or slower belt. Therefore, this study helps to address the role of error size and variance in locomotor adaptation, but not necessarily direction or timing of errors. A second limitation is that the study design allowed for the random blocked group to practice walking at only 32 different asymmetries, while the serial and random practice groups experienced up to 720 different asymmetries, with an equal amount of time where the variable limb belt was slower and faster than the constant limb belt. The randomization function used here and the boundaries placed on it may have made for an experience where the random blocked practice group walked more frequently at a faster velocity than a slow one, effectively making for more practice closer to the transfer asymmetries (Fig. 4). A third limitation was that subjects were not tested for recall or generalizability when the variable limb belt was slower than the constant belt, even though they received practice for about half of the acquisition phase at such an asymmetry. Turnham et al. (2012) have demonstrated that the order in which a gradual visuomotor perturbation is introduced to a learner ultimately affects how an adaptation to that task is retained. In this study the serial practice group was also given a gradual perturbation, where the belt velocities on the variable limb started slower than the constant limb, and increased gradually over time. If the findings by Turnham et al. (2012) are applicable to generalization of locomotor patterns, then it is possible that the serial practice group would be best suited to generalize to a walking context where the variable limb is moving slower than the constant one. Hence, these results cannot be extrapolated to generalizations to all possible split-belt walking asymmetries. Finally, all subjects did not walk at the same absolute velocities for each belt, but rather the velocities were a function of their measured SSWS. It is possible that subjects with a faster self-selected walking speed (SSWS) and shorter leg length may have been more challenged at high velocities compared to other individuals. However, there were no differences in SSWS or body height among groups; therefore the group-wide comparisons were not likely affected.

These findings highlight some differential mechanical strategies exhibited as a result of the acquisition experience during learning of a novel gait pattern. From these strategies, multiple future research directions may help to further clarify the role of sensory prediction errors on locomotor adaptation. First, certain gait parameters previously established as clear markers of predictive locomotor adaptation, such braking ground reaction force (GRF) (Mawase et al., 2013), were not as evident in the present study. However, previous studies have utilized a post-adaptation washout period on the treadmill, where the predictive adaptations are considered evident once a gait asymmetry is removed. The post-adaptation period was not measured in the present study. Future studies investigating the effect of variable practice on the ability to de-adapt may be better suited to utilize these parameters. Second, the study cohort in the present study represented a young, healthy population, and therefore the effect of variable practice may not be applicable to other populations such as the elderly or individuals with gait deficiencies. If these populations have a loss of somatosensory information (or a decreased ability to integrate and process it), the ability of these individuals to adapt to a novel gait pattern may be reduced (Bunday and Bronstein, 2009). In turn, future studies could help to clarify whether the variable practice effect demonstrated in the present study can positively affect locomotor adaptation in other populations. Finally, this study observed learning in an acute sense, with subjects being tested for transfer 24 h after a novel acquisition experience. It remains unknown how a novel gait pattern is adapted to and stored when individuals are given multiple bouts of practice over longer periods of time. It is possible that a repeated training intervention could be used as a rehabilitative tool, where populations with gait deficiencies could be trained to walk overground with a new locomotor pattern after frequent practice bouts.

In conclusion, this study investigated the effects of serial, random blocked, and random training conditions on locomotor adaptation to a novel gait. It was found that: (i) random practice, the most variable condition, naturally resulted in the most unique walking strategy during acquisition; (ii) few, if any, unique strategies emerge when the transfer context is close to that of acquisition (as demonstrated from the results of Transfer 1); (iii) random blocked practice resulted in unique changes in step length over time during Transfer 2. This suggests that perhaps a moderate level of contextual interference results in the most unique generalization strategies in walking contexts much different from that of acquisition. The findings of this study support the idea that error size and variance does affect an individual's ability to adapt to a novel gait pattern and can alter the mechanical strategies employed by these individuals when asked to generalize their acquisition experience.

## MATERIALS AND METHODS

### Recruitment

Forty-eight individuals between the ages of 18 and 50 years of age that were able to walk on a treadmill for up to 30 min were recruited for this study (Table 4). Subjects were excluded from participation if they self-reported any cardiopulmonary, neurological, acute (within 6 months) or chronic musculoskeletal injuries to the lower limbs, or if they had any previous experience in walking asymmetrically on a split-belt treadmill. The Human Subjects Research Committee at the University of Oregon and the Institutional Review Board approved all study protocols and all subjects provided written informed consent prior to enrollment.

### Study design and experimental protocol

**Fig. 5.**
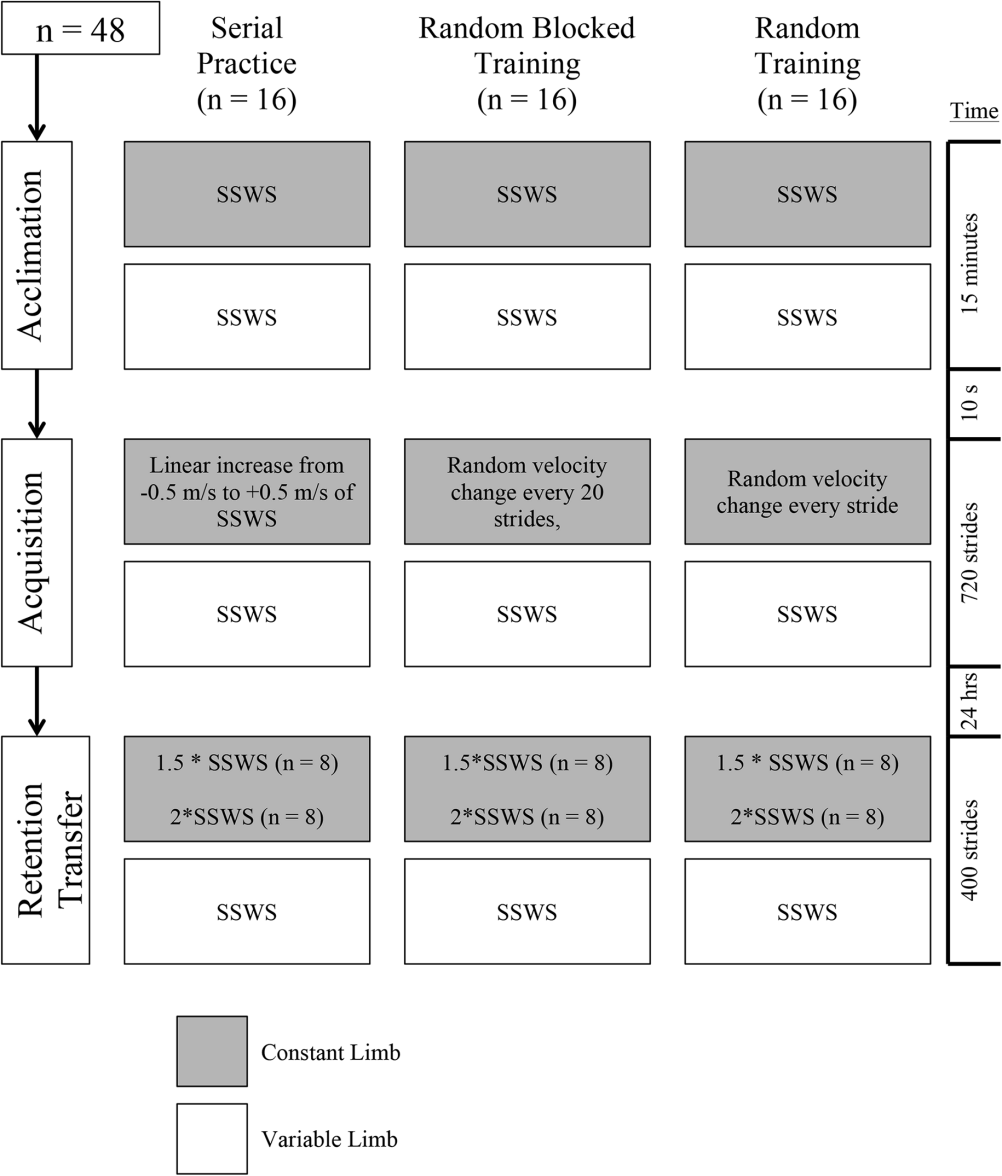
**Study design.** All subjects completed a 15-min acclimation phase with both belt velocities set to their SSWS. Following a 10-second pause subjects completed a 720-stride acquisition phase consisting of either serial, random blocked, or random practice. The serial practice group had their variable limb belt velocity increase linearly from −0.5 SSWS to +0.5 SSWS over the course of acquisition. The random blocked group had with their variable limb belt set to a random velocity within ±0.5 m/s of SSWS, and every 20 strides changed to a new velocity within ±0.5 m/s of SSWS and within ±0.5 m/s of the previous stride. The random group had their variable limb belt set to a random velocity for every stride within ±0.5 m/s of SSWS and ±0.5 m/s of the previous stride. All groups' constant limb was set to SSWS for all 720 strides of acquisition. 24 h later, half of the cohort completed a transfer test of 400 strides at a consistent 1.5:1 m/s (variable:constant) of SSWS asymmetry (Transfer 1), and the other half completed a transfer test of 400 strides at a consistent 2:1 m/s (variable:constant) of SSWS asymmetry (Transfer 2).

Each subject attended two consecutive days of experimental testing. On the first day, the average time across 4 trials it took a subject to walk 20 m overground was used to calculate SSWS. To ensure gait consistency during treadmill walking (Zeni and Higginson, 2010) and to collect biomechanical gait data during symmetric walking, subjects were then asked to walk for 15 min for an acclimation phase on an instrumented split-belt treadmill (Bertec, Columbus, OH, USA) where the velocity of both belts were tied to their SSWS. After 15 min, subjects completed one of three 720-stride acquisition protocols to which they were randomly assigned: serial, random blocked, or random practice. For all acquisition protocols, the non-dominant limb (constant limb) was consistently driven at SSWS (Fig. 5), while the dominant limb (variable limb) was driven according to the assigned practice protocol. Limb dominance was determined as the one the subject would prefer to use to kick a soccer ball.

For the variable limb, subjects in the serial practice group began with belt velocity set to SSWS−0.5 m/s on the first stride, and then the belt velocity increased linearly by 1/720 m/s on every subsequent stride so that on the 720th stride, variable limb belt velocity was SSWS+0.5 m/s. For the random blocked practice group, the variable limb belt began at a random velocity within ±0.5 m/s of SSWS, continued at that velocity for a block of 20 strides, and then randomly changed to a new velocity within ±0.5 m/s of SSWS and within ±0.5 m/s of the previous block. For the random practice group, the variable limb belt velocity changed randomly every stride within ±0.5 m/s of SSWS and ±0.5 m/s of the previous stride. The belt velocities were preset and organized with respect to practice protocol and SSWS, and then deployed by a custom-written MATLAB script (Mathworks, Natick, MA, USA) to automate control of the treadmill belts. This automation method has been previously established as a way to remove any effect of researcher error on changing belt velocities accurately on a step-by-step basis and ensure that treadmill belts were only accelerating during swing phase of gait as a way to prevent additional perturbations during walking (Hinkel-Lipsker and Hahn, 2016a).

Subjects were given 24 h to allow for consolidation of motor memories following the acquisition experience (Brashers-Krug et al., 1996) and for the adapted gait asymmetry to wash out through overground walking. Following the 24 h period, subjects were then asked to return and complete one of two transfer tests. One test, Transfer 1, was used to determine how well individuals from each practice group were able to generalize their acquired gait pattern to a context close to that of what was previously experienced. Thus, we used a constant 1.5:1 (variable:constant) belt velocity asymmetry over 400 strides for this test. Since the maximum belt velocity on the variable limb experienced by any participant during acquisition was SSWS +0.5 m/s, no subjects walked at a 1.5:1 asymmetry during this phase. Half of all the participants from each practice group completed this test. The other half completed a different test, Transfer 2, which was implemented in order to determine how well subjects from each practice group would apply their learned asymmetric gait to a context that was further away from the maximum SSWS +0.5 m/s velocity on the variable limb. Hence, a 2:1 (variable:constant) asymmetry was chosen as an asymmetry that had not been previously experienced but ensured that the subjects' variable limb was not moving so fast as to induce a running gait. For a full description of the ranges and distributions of variable limb belt velocities experienced by subjects, see Fig. 4.

### Data collection

Demographic data, including age, sex, height, and weight, were recorded on the first day of testing. Three-dimensional (3D) marker coordinate data were collected at 60 Hz from 54 reflective markers placed on participants' bony landmarks (Sawers and Hahn, 2012) using an 8-camera motion capture system (Motion Analysis, Santa Rosa, CA, USA). Additionally, GRF data were collected from two force plates underneath the two treadmill belts (Bertec, Columbus, OH, USA) at 1200 Hz. These data were synchronized with marker coordinate data using Cortex motion capture software (Motion Analysis, Santa Rosa, CA, USA), and were collected during the final 20 strides of acclimation, and throughout acquisition and transfer.

### Data analysis

Marker coordinate data were low-pass filtered using a 4th order Butterworth with a 5 Hz cut-off frequency, and GRF data were low-pass filtered using a 4th order Butterworth with a 45 Hz cut-off frequency (Sawers et al., 2013a). These data were used to build a 13-segment model with Visual 3D software (C-Motion, Germantown, MD, USA).

Specific spatiotemporal, kinematic, and kinetic variables were calculated for each limb (constant and variable) to help describe the mechanical strategies that each group adopted as a result of their practice experience. In the case of the spatiotemporal and kinetic data, variables were chosen because of their ability to describe specific gait strategies that people adopt during asymmetric and/or novel gait, as previously noted in the literature. Kinematic variables were calculated to observe the changes in lower-limb motion resulting from gait adaptation. Internal joint moments were estimated using an inverse dynamics approach and normalized to body weight. Five different windows of time were used for analysis: acquisition, early Transfer 1, late Transfer 1, early Transfer 2, and late Transfer 2. The acquisition time window represented all 720 strides. Due to the organization of the practice protocols, specific times during acquisition for each group could not be extracted for analysis because the variable limb belt velocities were not equal for each group. Similarly, gait behavior was not extracted for each velocity because the time during adaptation when a particular velocity was experienced was unique to each group. Therefore analysis of data during acquisition represented the overall gait strategy adopted, when the variable limb belt was at times both slower and faster than the constant limb belt. Since gait adaptation, at minimum, requires 12-15 strides (Prokop et al., 1995), early transfer windows were calculated as the average of the first 20 strides for each limb during this test. In this way, the early transfer window was representative of the immediate generalized response to a novel belt asymmetry during transfer. Late transfer windows included the last 20 strides of the transfer test. We have previously shown that some lower-limb kinetic adaptations are apparent early in an asymmetric walking bout, but do not persist until the end (Hinkel-Lipsker and Hahn, 2016b). Hence, analysis of gait mechanics during late transfer would show any persistent gait adaptations. The descriptions and justifications for the choice of each variable are provided below.

#### Spatiotemporal

##### Double support time (DS)

This value was calculated as the length of time in which both limbs are in contact with the ground. Two double support times were calculated; one when the constant limb was the leading limb (double support – constant limb) and one when the variable limb was leading (double support – variable limb). It has been previously demonstrated that as individuals adapt their gait pattern to a novel asymmetry, double support time becomes more symmetric between limbs (Reisman et al., 2005), indicating predictive control of this parameter (i.e. not dependent on spinal feedback mechanisms). Thus, regardless of whether the faster moving or slower moving limb is leading, double support times would be virtually equivalent if an individual has fully adapted their gait to that asymmetry. To visualize the level of double support symmetry over time, the ratio of double support time on the variable limb compared to the constant limb for each stride (Reisman et al., 2013) was calculated as:
(1)
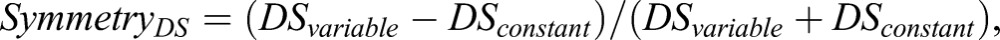
where *DS_variable_* is the variable limb time in double support, and *DS_constant_* is double support time for the constant limb.

##### Step length (SL)

This value was calculated as the anterior-posterior distance from the leading foot calcaneus making contact with the ground at heel strike to the trailing foot calcaneus. Similar to double support time, this metric has been previously demonstrated as one that is under predictive control (Reisman et al., 2005). The difference in step length between limbs decreases over time as a person adapts their gait, and it has been observed across many studies that even when treadmill belt asymmetry is held constant, subjects match their step length between limbs (e.g. Reisman et al., 2013; Roemmich and Bastian, 2015). Mean double support time and step length for each analysis window (acclimation, acquisition, Transfer 1, Transfer 2) were extracted as discrete values for statistical testing. Similar to double support symmetry, step length symmetry was calculated as (Reisman et al., 2013):
(2)
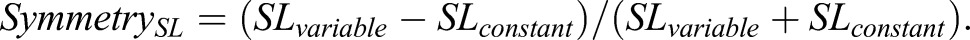


#### Kinetic

##### Anterior-posterior GRF

Peak braking force was measured as the minimum value for the GRF time series. This metric has been previously used as an indicator of predictive control of ankle stiffness, where more adapted individuals are better able to reduce braking force, increasing walking efficiency. In contrast, less adapted individuals have a higher braking force, slowing the forward velocity of the center of mass, at the expense of increased energy expenditure (Ellis et al., 2013; Mawase et al., 2013; Ogawa et al., 2014).

##### Hip extensor moment (HEM)

When measured during late swing phase (70-100% gait cycle), this metric indicates control of limb swing velocity, with associated increased energy absorption to slow the velocity of the swing leg (Winter and Rogers, 1992). It is likely that an adapted individual would control their leg swing velocity in a way where higher hip extensor energy absorption would not be necessary, thus minimizing energy expenditure, as higher leg swing velocity requires more work to be performed to slow it prior to heel strike (Doke et al., 2005).

##### Knee extensor moment (KEM)

When measured during stance phase, (0-60% gait cycle) the peak knee extensor moment can be used as a measure of loading asymmetry between the two limbs (Roemmich et al., 2012). Compared to using peak vertical GRF to measure limb loading, peak knee extensor moment gives a loading measure relative to the knee joint. It has been acknowledged previously that the knee extensors perform negative work during weight acceptance to prevent excess knee flexion from occurring (Kepple et al., 1997).

#### Kinematic

Sagittal-plane ankle, knee, and hip angles were calculated to provide descriptions of the overall motion of the lower limbs, and to determine if there were differences in that motion across practice groups.

##### Peak ankle dorsi- and plantar-flexion angles (ADA and APA)

Peak values were calculated for each stride from 30-65% GC as the maximum angle to eliminate extraction of a peak value that occurs early in the gait cycle (such as dorsiflexion during initial contact or plantar flexion during foot flat).

##### Peak knee flexion angle (KFA)

During stance this value was calculated as the minimum sagittal plane knee angle from 0-65% gait cycle (GC).

##### Peak hip flexion and extension angle (HFA and HEA)

These values were calculated as the maximum value during swing (65-100% GC) and minimum value during stance (0-65% GC). Each measurement was normalized to one gait cycle (1-100%), or the time from heel strike on one limb to the subsequent heel strike on the same limb. To perform statistical analyses, discrete peak values were calculated from each gait cycle and averaged to find the mean peak value across all strides for each discrete variable. Ensemble curves were also calculated to provide a qualitative time-series average for each parameter during each of the five time windows. Kinematic and kinetic calculations were performed using Visual 3D (C-Motion, Germantown, MD, USA), and variable extraction was performed using MATLAB (Mathworks, Inc., Natick, MA, USA).

### Statistical analysis

To analyze the effect of practice group on biomechanical gait variables for each limb, five two-way multivariate analyses of variance (MANOVAs, α=0.05) were run using SPSS v.23 (IBM, Armonk, NY, USA); one for each of the five time windows. Practice group (serial, random blocked, and random) and limb (constant and variable) were included as independent variables, and the ten aforementioned gait variables as dependent variables. Outliers and assumptions of univariate and multivariate normality, multicollinearity, homogeneity of covariance, and homogeneity of variance were tested for. In the case of several variables, outliers were present. These were corrected for through either square root or logarithmic transformations. The summary results of these particular data are presented in their raw form (Manikandan, 2010). If significant main effects or interactions were revealed, Bonferroni-adjusted pairwise comparisons were made. Gait behavior during the acclimation phase was included in all statistical tests to examine the difference between each practice group's gait behavior during acquisition and both transfer tests. Prior to statistical analysis, it was noted that one subject (Random Blocked – Transfer 2) had a SSWS of 1.62 m/s, and therefore the variable limb belt velocity during Transfer 2 was 3.24 m/s; a typical running velocity. To avoid the effect of having one walking limb and one running limb introduce an additional confounding variable, this subject's data were removed.
